# Investigation of Channel-Forming Activity of Polyene Macrolide Antibiotics in Planar Lipid Bilayers in the Presence of Dipole Modifiers

**Published:** 2014

**Authors:** S. S. Efimova, L. V. Schagina, O. S. Ostroumova

**Affiliations:** Institute of Cytology, Russian Academy of Sciences, Tikhoretsky Ave., 4, St. Petersburg, 194064, Russia

**Keywords:** planar lipid bilayers, polyene antibiotics, sterols, styryl dyes, sphingolipids, flavonoids, phospholipids

## Abstract

The role of membrane components, sterols, phospholipids and sphingolipids in the formation and functioning of ion-permeable nanopores formed by antifungal macrolide antibiotics, amphotericin B, nystatin and filipin in planar lipid bilayers was studied. Dipole modifiers, flavonoids and styryl dyes, were used as a tool to study the molecular mechanisms of polyene channel-forming activity. The introduction of dipole modifiers into the membrane bathing solutions was shown to change the conductance of single channels and the steadystate transmembrane current induced by polyene antibiotics in the sterol-containing phospholipid-bilayers. The conductance of single amphotericin B channels was found to depend on the dipole potential of the membrane. The experiments with various phospholipids, sterols, and polyenes led to the assumption that the shape of a phospholipid molecule, the presence of double bonds at the positions 7 and 22 of a sterol molecule, the number of conjugated double bonds, and the presence of an amino sugar in the polyene antibiotic molecule are important factors impacting the stability of polyene-lipid complexes forming ion-permeable pores. Experimental and literature data presented in the paper suggest that the channel-forming activity of polyene antibiotics is also affected by the physicochemical properties of polyene-enriched ordered membrane domains.

## INTRODUCTION

Polyene macrolide antibiotics are among the most effective drugs against fungal infections and deep systemic mycoses. They have been widely used in clinical medicine for many decades. Polyene macrolides also attract attention due to their anti-tumor and antiviral activity [1-3]. In spite of side effects, such as nephrotoxicity, anemia, and cardiac arrhythmia [4, 5], polyene macrolide remains the drug of choice for treatment of immunosuppressed patients [6, 7]. Pharmaceutical technologies develop innovative formulations that aim at reducing the concentration of free AMB in patients’ serum without compromising its therapeutic efficacy.

**Fig. 1:**
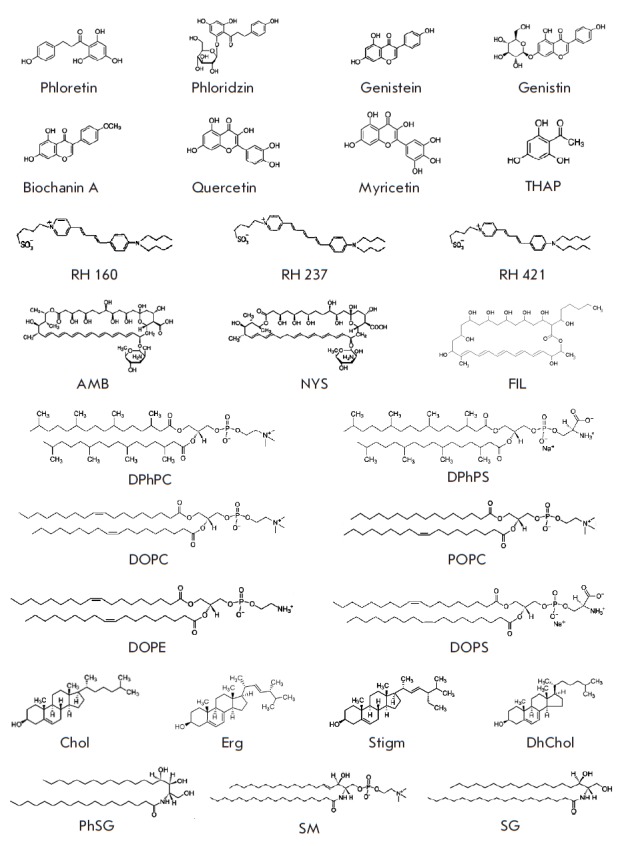
Chemical structures of flavonoids (phloretin, phloridzin, genistein, genistin, biochanin A, quercetin, myricetin and THAP), styryl dyes (RH 160, RH 237 and RH 421), polyenes (AMB, NYS and FIL), phospholipids (DPhPC, DPhPS, DOPC, POPC, DOPE and DOPS), sterols (Chol, Erg, DhChol and Stigm) and sphingolipids (PhSG, SM and SG)

The main representatives of non-aromatic polyene macrolide antibiotics are amphotericin B (AMB) [8], nystatin (NYS) [9, 10], and filipin (FIL) [11]. The lactone ring of amphotericin B contains 38 carbon atoms (*Fig. 1*). Hydrophilic and heptaenic chains in the AMB macrolactone ring include C_1_–C_15_ and C_20_–C_33_ carbon atoms, respectively. These chains are arranged parallel to each other. The C_20_–C_33_ heptaenic chain is a rigid system consisting of seven double bonds. The hydrophilic chain of AMB contains hydroxyl and carbonyl groups. The hydroxyl groups in the hydrophilic region of the molecule are arranged in one plane. A carboxyl group and a mycosamine residue are located at positions 6 and 19, respectively. Another hydroxyl group is located in the hydrophobic portion of the molecule at position 35. The chemical structure of nystatin, a tetraene, is similar to that of AMB. Nystatin differs from AMB in the positions of hydroxyl groups in the hydrophilic chain and discontinuity of the conjugated double bond system. A saturated bond divides the chromophore into the diene and tetraene portions. Filipin belongs to methylpentaens and has a smaller polyene fragment and no amino sugar residue as compared to AMB and NYS [12].

It is widely accepted that polyene antibiotics bind to the plasma membranes of the target cells, participate in formation of transmembrane pores and disrupt the water- electrolyte balance in cells, leading to cell death. The presence of sterols in the target cell membranes is a prerequisite for pore formation [8, 13, 14]. Despite 40 years of research into the molecular mechanisms of formation and functioning of the AMB channel, its precise molecular architecture is still under debate. Various models of AMB channels have been proposed. The most popular one is the sterol-dependent model, where the channel, in case of a two-sided (in respect to the membrane) addition of the antibiotic, is formed via association of two “half-pores” formed by polyene-sterol complexes located in opposite monolayers [8, 13, 15]. The cylindrical half-pore is formed by the same number (7 to 10) of antibiotic and sterol molecules, which are oriented perpendicular to the membrane plane. The cavity of the pores is lined with hydrophilic chains of lactone rings. A transmembrane pore is formed via hydrogen bonds between the hydroxyl groups of AMB molecules that are present in the interacting half-pores [12].

The sterol-dependent membrane activity of amphotericin B indicates that the therapeutic efficacy of AMB is primarily related to its differential preference for various sterols in cell membranes. It is well known that cholesterol (Chol) is the major membrane sterol in mammalian cells, whereas ergosterol (Erg) is the major sterol in fungi. It is still unclear whether the specificity of interaction of various polyenes with cell membranes is due to better stability of the AMB–Erg complex as compared to the AMB–Chol one or if the observed effects are mediated by different impacts of these sterols on structural and dynamic properties of membranes [16, 17].

**Fig. 2:**
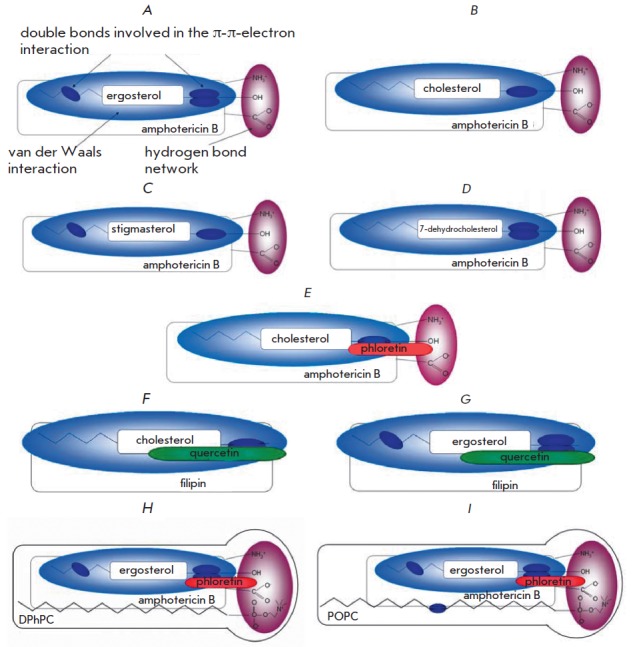
Schematic representation of intermolecular bonds in the complexes of AMB-Erg (*A*), AMB-Chol (*B*), AMB-Stigm (*C*), AMB-DhChol (*D*), AMB-Chol with phloretin (*E*), FILChol with quercetin (*F*), the FIL-Erg with quercetin (*G*), AMB-Erg-DPhPC with phloretin (*H*) and AMB-Erg-POPC with phloretin (*I*). AMB-Chol and AMB-Erg complexes as shown in [20] with some changes

The data reported by Neumann *et al*. [18, 19] support the first hypothesis. The more rigid and extended molecular shape of Erg, compared to Chol, facilitates Erg interaction with AMB. Since van der Waals interactions between rod-shaped molecules depend on their relative orientation and reach the maximum when two molecules lie in one plane and are parallel to each other, the π-π-electron interaction between a double bond in Erg side chain and AMB polyene chromophore may be an additional site required for stabilizing the proper orientation of the complex (*Fig. 2 A, B*) [20]. In case of Chol, not only is the energy of the complex formation higher (no double bond in the side chain of the sterol molecule), but in addition there is a need to compensate for entropic losses associated with a decrease in conformational flexibility of the sterol side chain. The results of the studies of mobility of AMB and sterols molecules in phospholipid bilayers by 2H NMR conducted by Matsumori et al. [21] confirmed the hypothesis of stronger intermolecular interaction between AMB and Erg compared to Chol.

Sterols are responsible for membrane fluidity and are predominantly located in the more ordered membrane domains, lipid rafts, which may be considered as an argument in favor of the second hypothesis. It has been shown that AMB has higher affinity for sterol-containing ordered phase and hence it can, like sterols, be accumulated in lipid rafts [17, 22]. Several studies indicate that the presence of AMB increases orderding of Chol-containing membranes and that no such effect is observed in case of Erg-containing membranes [17, 23, 24].

Czub and Baginski [17] demonstrated that the negatively charged carboxyl group (COO^-^) in AMB molecule is shifted towards the aqueous phase compared with the protonated amine group (NH3 +). The authors suggested that the dipole of the polar head of AMB (COO- → NH_3_^+^) tends to be oriented parallel to the dipoles of phosphatidylcholine polar heads, thus increasing the dipole potential of the membrane. This jump in the potential occurs at the bilayer–solution interface as a result of specific mutual orientation of the lipid membrane dipoles and the adjacent water dipoles [25-27] and plays an essential role in regulation of transport across the membrane.

As mentioned before, polyene macrolide antibiotics exhibit their antifungal effect by binding to membrane sterols, but little information is available on the role of other membrane components, in particular, phospholipids and sphingolipids. There is some evidence to suggest that phospholipids affect the activity of polyene antifungals. According to the published data, polyene antibiotics can form transmembrane pores in the bilayer even in the absence of sterols [28-32]. Fujii et al. [34] have shown that AMB can specifically interact with phospholipid molecules. In the ^2^H NMR studies of liposomes made of dimyristoyl-phosphatidylcholine with AMB, Dufourc *et al*. [34] have noted the improved ordering of acyl chains of this lipid molecule in its interaction with AMB. Furthermore, based on the analysis of circular dichroism spectra of AMB in liposomes in the absence of sterol, Balakrishnan and Easwaran [23] have suggested the presence of a multi-molecular organized structure in the bilayer, in which AMB interacts with acyl chains of the dipalmitoyl-phosphatidylcholine molecule at a 1:1 ratio. The differential scanning calorimetry studies by Fournier *et al*. [23] showed that AMB induces phase separation in the membrane; namely, that three phases of dipalmitoyl-phosphatidylcholine liposomes are simultaneously detected in the presence of AMB. The first phase corresponds to pure phospholipid, the second and the third phases are characterized by phase transition in a wide range of temperatures above the phase transition point of pure phospholipid. Furthermore, Paquet *et al*. [23] demonstrated a dose-dependent increase in the lipid phase transition point from gel to the liquid crystal state in the presence of AMB. Milhaud *et al*. [17] suggested that AMB interacts with multi-molecular phospholipid ensembles. The results obtained by Sternal *et al*. [17] using the molecular dynamics methods do not contradict the hypothesis of interaction between polar heads of AMB and dimyristoyl-phosphatidylcholine. Such interaction was observed, in particular, between the carboxyl group of AMB and the amino group of a lipid. Herec* et al*. [17] suggested that hydrogen bonds between the horizontally oriented AMB molecules and polar groups of a lipid lead to condensation of the bilayer.

We have found only indirect evidence of possible interactions between polyene macrolides and membrane sphingolipids. For example, Zager [40] showed that polyene antibiotics affect the concentration of phospholipids and ceramides in the plasma membrane. Nagiec *et al*. [41] found that a mutant strain of *Saccharomyces cerevisiae*, capable of growth without producing sphingolipids, is more susceptible to AMB than wild-type cells. Studies of the effect of the sphingolipid composition of a membrane on the activity of polyene macrolides are also interesting because sphingolipids, as well as sterols and polyenes, are located in lipid rafts [17].

The aim of the present work was to determine the molecular mechanisms of the formation of polyene transmembrane pores in membranes containing various phospholipids, sterols, and sphingolipids. Dipole modifiers, namely flavonoids and styryl dyes, which are capable of altering the dipole potential of membranes, were used as a research tool. The choice of dipole modifiers was based on their successful application in the studies focused on formation and functioning of ion channels in model and cell membranes [42-51].

## MATERIALS AND METHODS

**Materials**

The following reagents were used: KCl, HEPES, pentane, ethanol, chloroform, dimethyl sulfoxide (DMSO), hexadecane and squalene, phloretin, phloridzin, genistin, genistein, quercetin, myricetin, biochanin A, 2 ‘, 4’, 6’-trihydroxyacetophenone monohydrate (THAP), RH 421, amphotericin B (AMB), nystatin (NYS) and filipin (FIL) (Sigma, USA); RH 160 and RH 237 (Molecular Probes, USA); 1,2-diphytanoyl-* sn*-glycero-3-phosphocholine (DPhPC), 1,2-diphytanoyl-* sn*-glycero-3-phospho-l-serine (DPhPS), 1,2-dioleoyl-*sn*-glycero-3-phosphocholine (DOPC) 1-palmitoyl-2-oleoyl-*sn*-glycero-3-phosphocholine (POPC), 1,2-dioleoyl-*sn*-glycero-3-phospho-l-serine (DOPS), 1,2-dioleoyl-*sn*-glycero-3-phosphoethanolamine (DOPE), cholesterol (Chol), ergosterol (Erg), 7-dehydrocholesterol (DhChol), stigmasterol (Stigm), N-stearoyl-phytosphingosine from *S. cerevisiae* (PhSG), porcine brain sphingomyelin (SM), a synthetic sphingolipid N-stearoyl-D-*erythro*-sphinganine (SG) and 1,2-dipalmitoyl-*sn*-glycero-3-phosphoethanolamine- N-(lissamine rhodamine) (Rh-DPPE) (Avanti Polar Lipids, USA). Chemical structures of flavonoids, styryl dyes, polyenes, phospholipids, sterols and sphingolipids are shown in *Fig. 1*.

**Measurement of currents flowing through the planar lipid bilayers**

The bilayer lipid membranes were formed using the Montal and Mueller method [52] by combining the condensed lipid monolayers on the aperture in a Teflon film dividing the experimental chamber into two (*cis*and* trans-*) compartments. The volume of each compartment was 1.5 ml, the thickness of the Teflon film was 10 μm, and the aperture diameter was ~ 50 μm. Before the membrane formation process was started, the aperture in the Teflon film had been pretreated with hexadecane. Monolayers were formed on the air–water interface of the solution of 1 mg/ml lipid in pentane. The phospholipid:sterol and phospholipid:ergosterol: sphingolipid mixtures were used to form monolayers (the molar ratios were 67:33 mol % and 53:27:20 mol %, respectively). The channel-forming activity of polyenes was measured under the same ionic composition of aqueous electrolyte solution (2.0 M KCl). The acidity of the solution (pH 7.0) was maintained with a 5 mM HEPES-KOH buffer mixture.

Polyene antibiotics were added to the aqueous phase in both compartments: AMB and NYS as DMSO solution (10^-4^ to 10^-3^ M, respectively), and FIL as ethanol solution (10^-4^ M) to a final concentration of 10^-8^–10^-6^ M in the membrane bathing solution. The two-side administration of polyene antibiotics was chosen, because according to [8, 13, 15] the channels are formed by two associated half-pores. The final concentration of ethanol or DMSO in the chamber did not exceed 0.1% and did not cause any changes in the consistency of the membrane conductance.

Flavonoids phloretin, phloridzin, genistin, genistein, quercetin, myricetin, biochanin A and THAP were added in both compartments of the chamber as millimolar solutions in ethanol or DMSO to a final concentration of 20 μM in the membrane bathing solutions; and styryl dyes RH 160, RH 237, and RH 421, to a concentration of 5 μM.

The current flowing through the lipid bilayer membrane was measured in voltage clamp mode. Ag/AgCl electrodes with 1.5% agarose/2 M KCl bridges were used to apply the transmembrane voltage (*V*) and measure the transmembrane current. Positive voltage refers to the potential initiating flow of cations from the *cis*-compartment to the *trans*- one. Electrophysiological measurements were performed at room temperature.

Transmembrane currents were measured and digitized in a voltage clamp mode using Axopatch 200V and Digidata 1440A systems (Axon Instruments, USA). The data were processed using an 8-pole Bessel filter (Model 9002, Frequency Devices) and filtering frequency of 1 kHz. The data were processed using the Clampfit 9.0 software package (Axon Instruments, USA). Statistical analysis of the data was performed using Origin 8.0 software (OriginLab, USA).

The average ratio (*I*∞/*I*∞*^0^*) of the steady-state integral transmembrane current, induced by a channel-forming agent (AMB, NYS and FIL) in the presence (*I*∞) and in the absence (*I*∞*^0^*) of dipole modifiers, was defined as the arithmetic mean of *I*∞/*I*∞*^0^*measured in three to nine bilayers (mean ± SE). The steady-state number of channels operating in the membrane was defined as a ratio between the steady-state transmembrane current (*I*∞*^0^*) and the current flowing through a single channel (*i*).

Single-channel conductance (*g*) was defined as the ratio between the current flowing through a single channel (*i*) and the transmembrane potential difference (*V*). To construct histograms of current fluctuations, the transmembrane current values were determined by changes in the amplitude of currents at opening (or closing) of single channels. The total number of events (*N*) used for the analysis at a fixed value of the transmembrane potential ranged from 100 to 5000. Relative frequencies of the transmembrane current values are plotted along the *Y *axis . All peaks in the histograms were approximated using the normal density distribution. The distribution hypothesis was verified using χ2 (P < 0.05).

**Measurements of channel selectivity**

To measure the cation-anion selectivity of the channel, a 10-fold concentration gradient of KCl electrolyte has been created in the membrane. The selectivity of AMB channels was measured at solution concentrations of 0.2 and 2.0 M KCl in the *cis- *and *trans-*compartments of the experimental chamber, respectively. The anion transference number (*t*^-^) (*t*^-^ + *t*^+^ = 1) was calculated using the Henderson equation [53]:

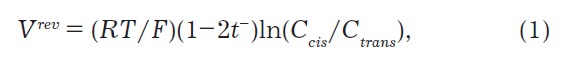



where *V*^rev^ is the reversal potential corresponding to zero transmembrane current at a given ratio between concentrations of ions penetrating from the *cis- *and* trans*-sides of the membrane (*C*_cis_/*C*_trans_); *R *is the universal gas constant (*R *= 8.31 J/(mol K)); *T *is thermodynamic temperature (*T *= 294 K); and *F *is the Faraday constant (*F *= 96485 C/mol).

**Confocal microscopy of giant unilamellar vesicles**

Giant unilamellar vesicles were produced by electroformation using Nanion Vesicle Prep Pro workstation (Germany) (standard protocol, 3 V, 10 Hz, 1 h, 25°C). The lateral phase separation was visualized by introducing a fluorescent Rh-DPPE probe into the source lipid solution of POPC in chloroform (11 mM). Rh-DPPE concentration in the sample was 1 mol %. The resulting liposome suspension was divided into aliquots. An aliquot without AMB was used a control. The experimental samples contained 100 or 300 μM of AMB. Vesicles were observed through immersion lenses 100.0×/1.4 HCX PL on an Apo Leica TCS SP5 confocal laser system (Leica Microsystems, Germany). The preparations were studied at 25°C. Rh-DPPE was excited by 543 nm light (He-Ne laser). It is known that Rh-DPPE in a bilayer with phase separation is preferably incorporated into the disordered liquid phase [54], while the ordered liquid and solid (gel) phases remain unstained [55]. At least four independent experiments have been performed for each system.

## RESULTS AND DISCUSSION

**The influence of dipole modifiers on conductance of single amphotericin channels**

**Fig. 3:**
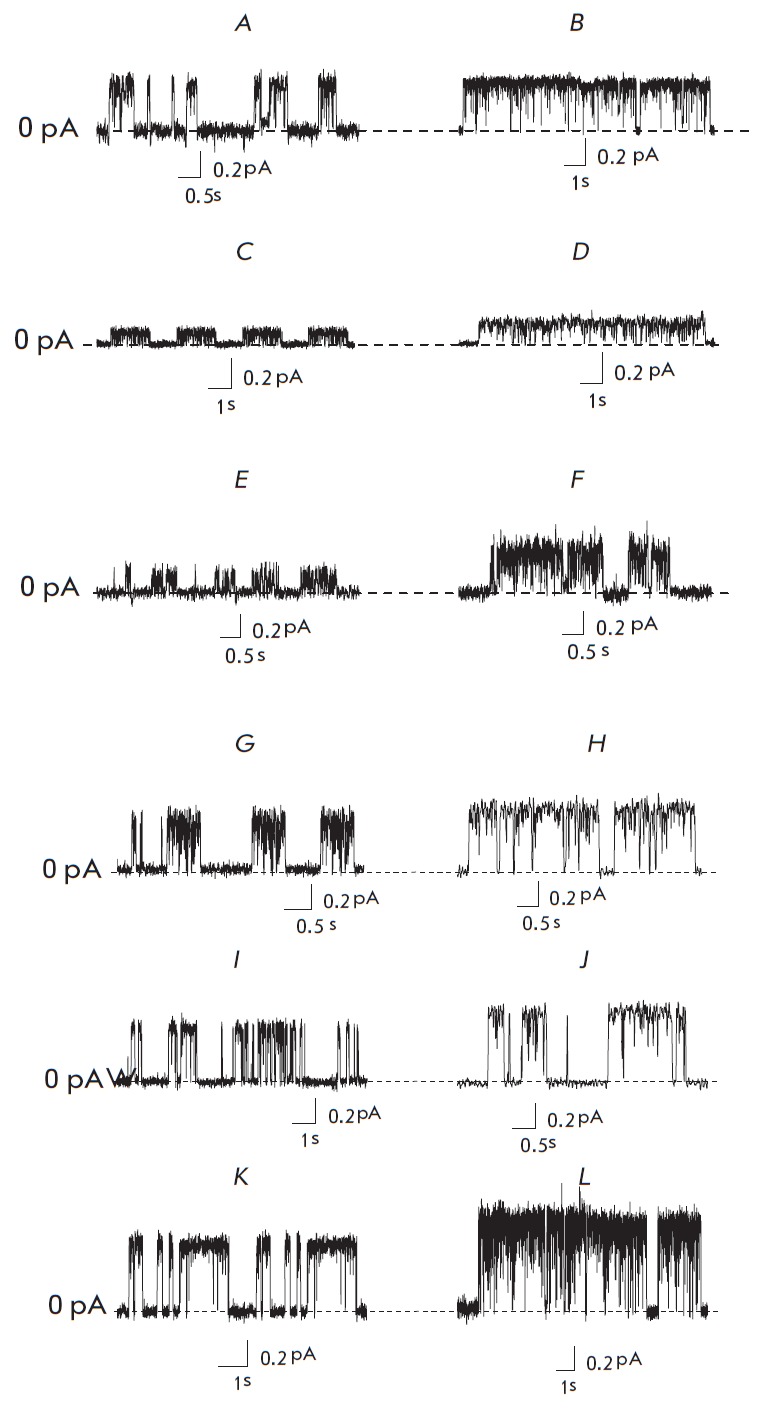
Fluctuations of transmembrane current through the single AMB channels in the lipid bilayers. Membranes were made from DPhPC:Chol (67:33 mol %) and DPhPC: Erg (67:33 mol %) and bathed in 2.0 M KCl (pH 7.0).* A*, *B *– control (no dipole modifiers). Membrane bathing solution contains (μM): 20 phloretin (*C*, *D*), 20 quercetin (*E*, *F*), 5 RH 160 (*G*, *H*), 5 RH 237 (*I, J*), 5 RH 421 (*K*, *L*). Dashed lines correspond to 0 pA. *V *= 50 mV

*Figure 3 *shows the examples of fluctuations in transmembrane current flowing through the single AMB channels in DPhPC:Chol membranes (*Fig. 3, left column)* and DPhPC:Erg bilayers (*Fig. 3, right column*), before and after the addition of dipole modifiers, flavonoids (phloretin and quercetin) and styryl dyes (RH 160, RH 237 and RH 421). *Figure 3 A, B *shows that the current flowing through single AMB channels in the absence of dipole modifiers does not depend on sterol type (Chol or Erg) in the membrane. The addition of phloretin to the membrane bathing solutions reduces the transmembrane current through single AMB channels, both in DPhPC:Chol membranes and in DPhPC: Erg bilayers (*Fig. 3 C, D*). However, the addition of quercetin reduces the transmembrane current through AMB channels only in DPhPC:Chol membranes but does not affect the transmembrane current through AMB channels in DPhPC:Erg membranes (*Fig. 3 E, F*). The introduction of styryl dyes of the RH series into the membrane bathing solutions increases the transmembrane current through single AMB channels. The current rises in the series RH 160 < RH 237 < RH 421 both for DPhPC:Chol membranes (*Fig. 3 G, I, K*) and for DPhPC:Erg bilayers (*Fig. 3 H, J, L*).

*Table 1 *presents the ratios between conductance of single AMB channels in the absence and in the presence of dipole modifiers at the transmembrane potential of 50 mV (*g*/*g_V=50_*). The results shown in *Table 1* reveal that phloretin reduces the conductance of single AMB channels in DPhPC:Chol and DPhPC:Erg membranes by a factor of 3 and 2, respectively. However, the addition of quercetin reduces the conductance of single AMB channels by a factor of 1.7 in DPhPC:Chol membranes but basically does not change *g *in the case of DPhPC:Erg bilayers. The introduction of other flavonoids to the membrane bathing solutions, such as phloridzin, biochanin A, THAP, genistin or genistein, do not practically affect the conductance of AMB channels. The addition of styryl dye to the membrane bathing solutions increases *g *in the series RH 160, RH 237 and RH 421 in DPhPC:Chol membranes by factors of 1.3, 1.4 and 1.5 and in DPhPC:Erg bilayers by factors of 1.6, 1.7 and 2.1, respectively.

**Table 1:** Ratios between the conductances of single amphotericin channels in the absence and in the presence of various dipole modifiers at V = 50 mV (g/g_V=50_). Membranes were made from DPhPC:Chol (67:33 mol %) and DPhPC: Erg (67:33 mol %) and bathed in 2.0 M KCl (pH 7.0)

Dipole modifier		Membrane-forming solution	
		DPhPC:Chol	DPhPC:Erg
Flavonoid	Phloretin	3.30 ± 0.21	2.20 ± 0.41
	Phloridzin	1.00 ± 0.10	1.00 ± 0.10
	Quercetin	1.72 ± 0.21	0.95 ± 0.15
	Genistein	0.98 ± 0.09	-
	Genistin	0.96 ± 0.08	-
	Biochanin A	0.89 ± 0.11	-
	THAP	0.91 ± 0.15	-
Styryl dyes	RH 421	0.69 ± 0.07	0.49 ± 0.06
	RH 237	0.71 ± 0.08	0.61 ± 0.05
	RH 160	0.80 ± 0.09	0.63 ± 0.06

*Table 2 *presents changes in the dipole potential of DPhPC:Chol and DPhPC:Erg bilayers in the presence of dipole modifiers in the membrane bathing solutions. For example, phloretin reduces the dipole potential of DPhPC:Chol membranes by 75 ± 10 mV, and that of DPhPC:Erg bilayers by 150 ± 5 mV. The addition of quercetin to the membrane bathing solutions leads to an almost identical reduction in φd in Chol- and Erg-containing DPhPC-bilayers by 100 ± 15 mV. The introduction of genistin and THAP into the membrane bathing solutions has little effect on the φd of DPhPC: Chol and DPhPC:Erg bilayers. The addition of styryl dyes of the RH series to the membrane bathing solutions increases the dipole potential of the membrane. The ability to increase the dipole potential of sterol- containing membranes increases in the series RH 421 ≈ RH 160 < RH 237. The addition of styryl dye RH 237 to the membrane bathing solutions increases φd of sterol-containing bilayers by 80 ± 10 mV, regardless of sterol composition of the membrane. Meanwhile, the presence of RH 421 or RH 160 in the membrane bathing solutions increases the dipole potential of DPhPC:Chol and DPhPC:Erg membranes by 55 ± 10 mV and 50 ± 10 mV, respectively. Comparison of the values in *Tables 1 *and *2 *suggests a correlation between the changes in the conductance of single AMB channels and the dipole potential of sterol-containing DPhPC-bilayers upon introduction of dipole modifiers. These results suggest that the change in *g *with introduction of phloretin or styryl dyes into the solutions bathing the Chol- and Erg-containing DPhPC-membranes and quercetin into the solutions bathing the DPhPC:Chol bilayer may be associated with a change in the membrane dipole potential. The discrepancies between the changes in the conductance of single AMB channels and dipole potential of sterol-containing DPhPC-bilayers caused by introduction of dipole modifiers suggest that the change in *g *is caused not only by the changes in the membrane dipole potential, but may also result from interaction of dipole modifiers (phloretin, quercetin and/or styryl dyes) with the AMB-Chol and/or AMB-Erg complexes.

**Table 2:** Changes in the dipole potential (Δφ_d_, mV) of DPhPC:Chol (67:33 mol %)* or DPhPC:Erg (67:33 mol %)* membranes in the presence of different dipole modifiers

Dipole modifier		Membrane-forming solution	
		DPhPC:Chol	DPhPC:Erg
Flavonoid, 20 μM	Phloretin	-75 ± 10	-150 ± 5
	Phloridzin	-45 ± 10	-50 ± 10
	Quercetin	-110 ± 10	-105 ± 15
	Genistein	-35 ± 5	-40 ± 10
	Genistin	-30 ± 5	-
	Biochanin A	-75 ± 15	-80 ± 15
	THAP	-40 ± 10	-40 ± 10
Styryl dyes, 5μM	RH 421	50 ± 8	57 ± 9
	RH 237	75 ± 10	85 ± 5
	RH 160	55 ± 10	45 ± 5

* Results are taken from [69].

*Fig. 2 *shows schematic representation of intermolecular bonds in the polyene-sterol complexes. It is known that polyene-sterol complexes are formed by van der Waals interactions [19]. The strength of interaction in this case depends on the coplanarity and parallelism of polyene and sterol molecules. The relative orientation of molecules occurs through the formation of hydrogen bonds between the OH group of the sterol molecule and the amino sugar of the polyene molecule. The presence of extra (compared with Chol) double bonds in the steroid core and in the side chain of the Erg molecule leads to the formation of a stable AMB-Erg complex through additional points of π-π-electron interaction compared to AMB-Chol complex [20] (*Fig. 2 A, B). *Therefore, AMB-Erg and AMB-Chol complexes may interact with the dipole modifiers in a different manner.

## INFLUENCE OF DIPOLE MODIFIERS ON MULTICHANNEL MEMBRANE CONDUCTANCE INDUCED BY POLYENE ANTIFUNGALS

**Influence of sterol composition**

**Fig. 4:**
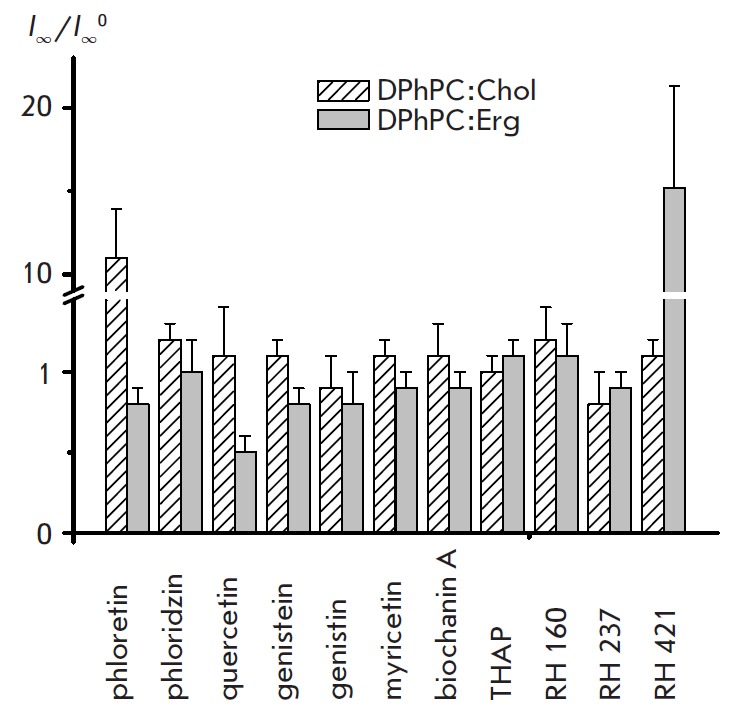
The ratio between the steady-state transmembrane currents induced by amphotericin B in sterol-containing bilayers before and after the introduction of various dipole modifiers (*I*∞/*I*∞^0^). Membranes were made from DPhPC:Chol (67:33 mol %) or DPPhC:Erg (67:33 mol %) and bathed in 2.0 M KCl (pH 7.0)

To test the hypothesis about the interaction between dipole modifiers and polyene-sterol complexes, we have studied the effect of dipole modifying agents on the steady-state transmembrane current induced by amphotericin B. The mean ratio between the steadystate transmembrane currents induced by AMB in Chol- and Erg-containing DPhPC bilayers before and after the introduction of various dipole modifiers (*I*_∞_*/I*∞*^0^*) at a transmembrane voltage of 50 mV is presented as diagram in *Fig. 4. *The addition of phloretin to the membrane bathing solutions caused a significant increase in the steady-state transmembrane current induced by AMB in DPhPC:Chol bilayers. No such influence of phloretin on *I*_∞_ was observed in case of DPh- PC:Erg membranes. The introduction of quercetin to the membrane bathing solutions does not affect *I*_∞_ in DPhPC:Chol membranes and reduces *I*_∞_ in DPhPC:Erg bilayers. Such flavonoids as phloridzin, genistein, genistin, biochanin A, myricetin and THAP do not impact *I*_∞_ in Chol- and Erg-containing DPhPC-membranes. The introduction of RH 421 to the solution bathing DPhPC: Chol bilayers does not practically affect *I*_∞_, and the addition of the modifier to the solution bathing DPh- PC:Erg membrane increases *I*∞. However, other styryl dyes, such as RH 160 and RH 237, have no effect on the multi-channel activity of AMB in Chol- and Erg-containing DPhPC membranes. It seems likely that in the case of the less energetically favorable AMB-Chol complex, phloretin, due to its “hairpin” conformation, can become a mediator between the polyene and sterol molecules and can stabilize the AMB-Chol complex (*Fig. 2 E*), which is expressed as an increase in AMB channel-forming activity in the presence of this dipole modifier in Chol-containing membranes. Quercetin, due to the greater depth of immersion into the bilayer compared with phloretin [56], can compete with sterols for the interaction with AMB and destabilize the most energetically favorable AMB-Erg complex, which expressed by a reduction of channel-forming activity of polyene (*Fig. 4*). Considering the fact that RH 421 styryl dye is characterized by intermediate chromophore immersion depth in the bilayer compared with RH 160 and RH 237 and has near-normal orientation on the membrane surface [57], we can assume its colocalization with the AMB-Erg complex. In this case, styryl dyes can be regarded as a third party to van der Waals interactions, which acts as an additional orienting factor via its participation in the π-π-electron interactions.

Since the main difference between Erg and Chol molecules is the presence of two double bonds at the position 7 in the steroid core and at the position 22 in the side hydrocarbon chain, the choice of sterols included those that differ from cholesterol in having one double bond at the position 7 or 22, 7-dehydrocholesterol (DhChol) and stigmasterol (Stigm), respectively (*see Fig. 1*). It has been determined that the addition of phloretin to the membrane bathing solution leads to a greater increase in the steady-state transmembrane current through DPhPC:Stigm membranes (*I*∞/*I*∞*^0^*= 5.3 ± 3.1), compared with DPhPC:DhChol bilayers (*I*∞*/I*∞*^0^*= 1.7 ± 0.3). The strength of phloretin effect on AMB-modified DPh- PC:Chol and DPhPC:Stigm membranes, as well as its absence in DPhPC:Erg bilayers and its weakness in the case DPhPC:DhChol membranes, indicates similar geometry of AMB-Chol and AMB-Stigm complexes and AMB-Erg and AMB-DhChol complexes, respectively. Schematic representation of intermolecular bonds in AMB-DhChol and AMB-Stigm complexes is shown in* Fig. 2 C, D*. Since the similarity between Chol and Stigm molecules and DhChol and Erg molecules is the absence or presence of a double bond at the position 7, respectively, one can suggest that the decisive role of electrical density distribution in the steroid core area (near the position 7), which can affect the possible formation of a hydrogen bond between the hydroxyl group of the sterol molecule and the OH groups of phloretin. The introduction of RH 421 to the solutions bathing DPhPC:Dh- Chol and DPhPC:Stigm bilayers does not significantly effect on *I*∞ (*I*∞/*I*∞* 0 *= 1.1 ± 0.1). As RH 421 is only effective in case of DPhPC:Erg membranes and does not affect AMB-modified DPhPC:DhChol bilayers, and Erg differs from DhChol in the presence of a double bond at the position 22, the results suggest that RH 421 is a more sensitive tool for studying AMB-sterol complexes than phloretin, and the double bond at position 22 still has an effect on the geometry and energy of the complex.

**Influence of the type of polyene antibiotic**

**Fig. 5:**
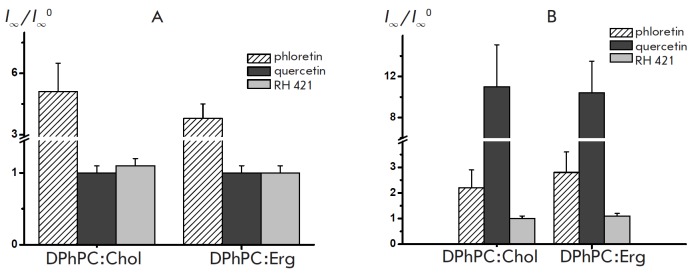
The ratios between the steady-state transmembrane currents induced by nystatin (*A*) and filipin (*B*) in sterol-containing bilayers before and after the introduction of various dipole modifiers (*I*∞/*I*∞^0^). Membranes were made from DPhPC:Chol (67:33 mol %) or DPPhC:Erg (67:33 mol %) and bathed in 2.0 M KCl (pH 7.0)

Since polyene molecules can also interact with dipole modifiers, their effect on the steady-state transmembrane current induced by nystatin and filipin (*Fig. 1*) has been studied in sterol-containing bilayers. NYS molecule differs from AMB molecule in the absence of a double bond in the middle of the polyene fragment, which may affect π-π-electron interactions in polyene- sterol complexes. FIL molecule, unlike AMB and NYS, does not contain an amino sugar residue. This structural difference should affect the formation of the network of hydrogen bonds between the polyene and sterol molecules. In the case of nystatin, *I*∞ increases with addition of phloretin both in DPhPC:Chol and in DPhPC:Erg containing membranes, while introduction of quercetin does not affect *I*∞ if the membrane contains either Chol or Erg (*Fig. 5 A*). Both flavonoids (phloretin and quercetin) increase the steady-state equilibrium transmembrane current induced by filipin, regardless of the type of the membrane-forming sterol, by a factor of 2 and 10 for DPhPC:Chol and DPhPC:Erg membranes, respectively (*Fig. 5 B*). However, the addition of RH 421 to the solutions bathing DPhPC:Chol and DPh- PC:Erg membranes does not change the steady-state transmembrane current induced by both nystatin and filipin (*Fig. 5 A, B*). Disruption of double bond conjugation in NYS molecule may destabilize the polyene-sterol complex and increase the depth of sterol immersion in the bilayer, pushing it away from the polar “head” of polyene molecules. Phloretin may be able to stabilize such NYS-sterol complexes. The absence of amino sugars in filipin molecule changes the hydrogen bond network in the polyene-sterol complexes and destabilize them. It is possible that localization of quercetin in the hydrocarbon region of the bilayer allows its interaction with a more hydrophobic polyene filipin (*Fig. 2 F, G*), thus significantly increasing the steady-state transmembrane FIL-induced current in the Chol- and Erg-containing DPhPC-bilayers.

**Influence of phospholipid composition**

**Fig. 6:**
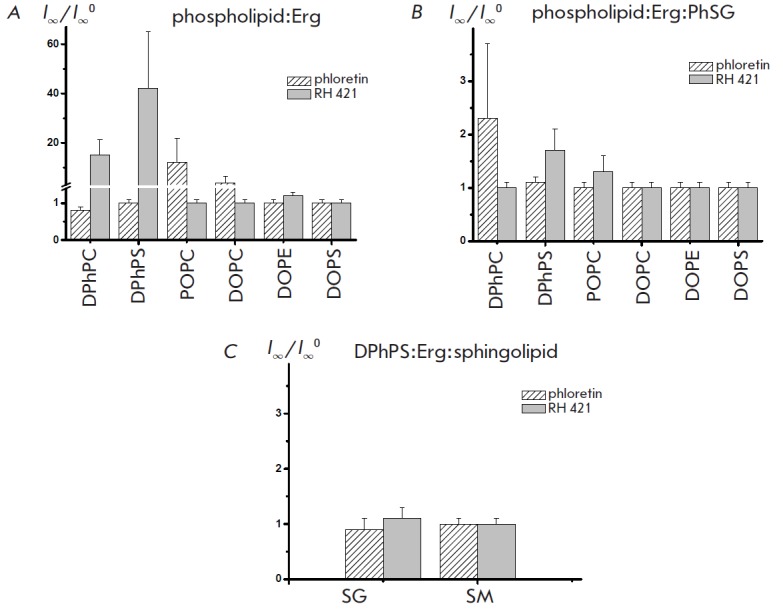
The ratios between the steady-state transmembrane currents induced by amphotericin B in ergosterol- containing bilayers before and after the introduction of the dipole modifiers (*I*∞/*I*∞^0^). Membranes were made from phospholipid: Erg (67:33 mol %) (*A*), phospholipid:Erg:PhSG (53:27:20 mol %) (*B*) and DPhPS:Erg:sphingolipid (53:27:20 mol %) (*C*) and bathed in 2.0 M KCl (pH 7.0)

The channel-forming activity of AMB in lipid bilayers that comprise, in addition to sterols, various phospholipids and sphingolipids was studied in the presence of phloretin and RH 421 to investigate the interaction of polyenes with other membrane components. The average ratio between the steady-state transmembrane currents induced by AMB in Erg-containing phospholipid bilayers before and after the introduction of dipole modifiers (*I*∞*/I*∞*^0^*) at a transmembrane voltage of 50 mV is shown in *Fig. 6 A*. It has been established that the introduction of phloretin into the membrane bathing solution leads to a significant increase in the AMB channel-forming activity in Erg-containing POPC (12- fold) and DOPC (4-fold) bilayers, while this dipole modifier does not affect the AMB-modified Erg-containing membranes formed with by DPhPC, DPhPS, DOPE and DOPS. The introduction of RH 421 to the membrane bathing solutions causes a manifold increase in *I*∞ through Erg-containing DPhPC (15-fold) and DPhPS (42-fold) bilayers, but does not affect the steady-state transmembrane current induced by AMB in Erg-containing membranes, including POPC, DOPC, DOPE and DOPS. Given that DPhPC, DPhPS, DOPE and DOPS molecules have a conical shape and DOPC and POPC molecules have a cylindrical shape [58, 59], we can assume that the latter are better fit for a rigid AMB molecule. Schematic representation of intermolecular bonds in AMB-Erg-DPhPC and AMB-Erg-POPC complexes is shown in *Fig. 2 H, I*. It is possible that strong “polyene-phospholipid” interaction weakens “polyene- ergosterol” interaction. Such polyene-sterol complex can be stabilized by phloretin molecules, which, due to their high conformational mobility and four functional hydroxyl groups, are able to act as intermediaries in the formation of hydrogen bond network between sterol and AMB. Differences between rigid rod-shaped AMB molecules and conical phospholipids (DPhPC, DPhPS, DOPE and DOPS) prevent strong “polyene-phospholipid” interaction and therefore there is no destabilization of the polyene-sterol complexes. Previously, we have assumed that RH 421 increases the channel-forming activity of AMB in Erg-containing DPhPC-membranes as this dipole modifier takes part in both hydrogen bonds network and in π-π-electron interactions between Erg and AMB molecules. Probably, similar processes take place in AMB-modified Erg-containing DPhPS bilayers.

**Influence of sphingolipid composition**

The introduction of sphingolipids into the membrane- forming solutions significantly affects the interaction between AMB molecules and phospholipids. It was established that phloretin is responsible for a 2-fold increase in *I*_∞_ in case of DPhPC:Erg:PhSG membranes and that RH 421 leads to a 1.7-fold increase in case of DPhPS:Erg:PhSG bilayers. Replacement of sphingolipid (PhSG to SG or SM (*Fig. 6 C*)) or phospholipid (DPhPC to DPhPS, POPC, DOPC, DOPE or DOPS in case of phloretin and DPhPS to DPhPC, POPC, DOPC, DOPE, DOPS in the case of RH 421 (*Fig. 6 B*)) component in the aforementioned mixtures does not increase *I*_∞_ in the presence of dipole modifiers. These results indicate that the sphingolipids introduced to the membrane-forming solution play an important role in the interaction of AMB with phospholipids and sterols.

**Fig. 7:**
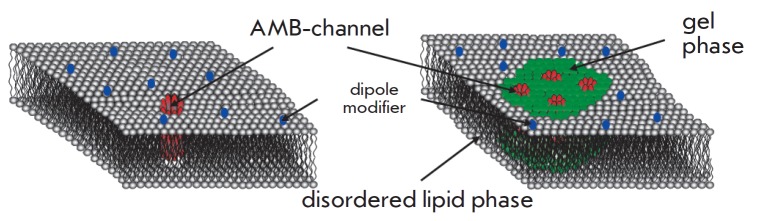
Schematic representation of the microenvironment of AMB channels in membranes with different concentrations of polyene antimycotics, corresponding to the functioning of single channels (*A*) and integral multi-channel current (*B*). High concentrations (*B*) of AMB provoke the formation of a more ordered lipid phase in the membrane (shown in green)

Since phloretin reduces the conductance of single AMB channels in DPhPC:Erg membranes, the lack of effect of this dipole modifier on the steady-state transmembrane current induced by AMB must indicate a jump in the number of open AMB channels. Lack of evidence for such a conclusion suggests two hypotheses: 1) differences in the properties of single AMB channels, in particular, the lack of selectivity of AMB channels responsible for integral current; in this case the conductance of the channels should not be a function of the membrane dipole potential; 2) differences in properties of the channel microenvironment in the membrane, i.e. single AMB pores and channels that are responsible for integral current are localized in the membrane regions with different properties, including those with different values of the membrane dipole potential (*Fig. 7*).

**Cation-anion selectivity of amphotericin channels**

To test the first hypothesis, we have measured the cation- anion selectivity of the single AMB channel and the integral transmembrane current. The results showed that AMB channels in sterol-containing bilayers are predominantly anion-selective, regardless of the degree of modification of the AMB membrane. The anion transference number for the single AMB channels* t*- = 0.9 ± 0.1, while *t*- for the integral current induced by AMB is 0.8 ± 0.1. The data obtained indicate that due to the high selectivity the conductance of the AMB channels that are responsible for integral current is expected to depend on the membrane dipole potential.

**Amphotericin channels in membranes with phase separation**

There is some evidence in favor of the second hypothesis. For example, a dose-dependent increase in the phase transition point of lipids from the gel phase to the liquid crystal one has been demonstrated in the presence of AMB [23]. It implies that AMB triggers the formation of a more ordered phase in the membrane. Moreover, as mentioned earlier, AMB molecules have higher affinity to ordered lipid domains (rafts) [17]. Since ordered lipid domains are rich in sphingolipids and sterols, their physicochemical properties are defined by the lipid composition of the membrane. It is known that the degree of ordering of lipid molecules and the likelihood of rafts formation depend on the type of sterol incorporated into bilayer [60-62]. Sphingolipid composition of the membrane is also important. In particular, PhSG is different from SG in having a hydroxyl group at the C_4_ position. Idkowiak-Baldys et al. demonstrated that C_4_-hydroxylation significantly affects the physical and structural properties of lipid microdomains [63]. The additional hydroxyl group most likely promotes condensation of lipid molecules by increasing the number of hydrogen bonds [64]. Plasma membranes of fungal cells contain phytosphingosine and ergosterol [65], and plasma membranes of mammalian cells contain sphingomyelin and cholesterol [66]. Evolutionary preference for these combinations can be attributed to the properties of ordered lipid domains formed by them. Moreover, some phospholipids with a low melting temperature, which are not localized in the ordered membrane domains, can induce the formation of these domains. Therefore, the ability to stabilize lipid rafts depends on phospholipid structure and decreases for the series DPhPC, DPhPS, POPC (DOPC) [67].

Fluorescent confocal microscopy of giant unilamellar vesicles demonstrated that dipole modifiers affect phase separation in liposomes [68]. Flavonoids biochanin A and phloretin lead to liquefaction of solid crystalline regions in liposome membranes and promote the formation of membrane rafts, while myricetin causes bilayer condensation. The data obtained by differential scanning calorimetry confirm the effect of flavonoids on phase separation in liposomes [68].

**Fig. 8:**
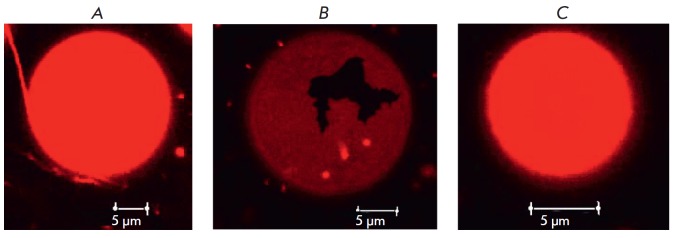
Photomicrographs of unilamellar POPC vesicles in the absence of polyene antibiotics (*A*) and in the presence of 300 rKM AMB in the membrane bathing solutions (*B*) and a combination of 300 rKM AMB and 400 rKM phloretin (*C*)

Our results show that liposomes from POPC are homogeneously stained in the absence of AMB; lateral heterogeneity of the membrane is not observed (*Fig. 8 A*). The addition of 300 μM AMB induces the formation of unstained dendritic domains in liposomes, which can be attributed to the solid lipid phase (*Fig. 8 B*). Phloretin in a concentration of 400 μM liquefies gel domains in AMB-modified vesicles and liposomes become homogeneously dyed (*see Fig. 8 C*). These data indicate that dipole modifiers influence the formation and dynamics of polyene-enriched ordered membrane domains.

## CONCLUSIONS

It has been established that the channel-forming activity of polyene antibiotics in lipid bilayers is defined by the superposition of several factors: the membrane dipole potential, stability of the polyene-lipid complexes, and physicochemical properties of ordered lipid domains.

## Abbreviations

AMB: amphotericin B
NYS: nystatin
FIL: filipin
DPhPC: 1,2-diphytanoyl-sn-glycero-3-phosphocholine
DPhPS: 1,2-diphytanoyl-sn-glycero-3-phospho-l-serine
DOPC: 1,2-dioleoyl-sn-glycero-3-phosphocholine
POPC: 1-palmitoyl-2-oleoyl-sn-glycero-3-phosphocholine
DOPS: 1,2-dioleoyl-sn-glycero-3-phospho-l-serine
DOPE: 1,2-dioleoyl-sn-glycero-3-phosphoethanolamine
Rh-DPPE: 1,2-dipalmitoyl-sn-glycero-3-phosphoethanolamine-N-(lissamine rhodamine)
Chol: cholesterol
Erg: ergosterol
DhChol: 7- dehydrocholesterol
Stigm: stigmasterol
PhSG: N-stearoyl-phytosphingosine (Saccharomyces cerevisiae)
SM: porcine brain sphingomyelin
SG: N-stearoyl-D-erythro-sphinganine
